# Supplementary material for: Amino Acid Substitution within Seven-Octapeptide Repeat Insertions in the Prion Protein Gene Associated with Short-Term Course
Source: Viruses. 2022 Oct 13;14(10):2245. doi: 10.3390/v14102245 (PMC9609758; doi:10.3390/v14102245)
Supplement: Supplementary file 1 [file viruses-14-02245-s001.zip › viruses-1887165-supplementary.pdf]

Supplementary Materials

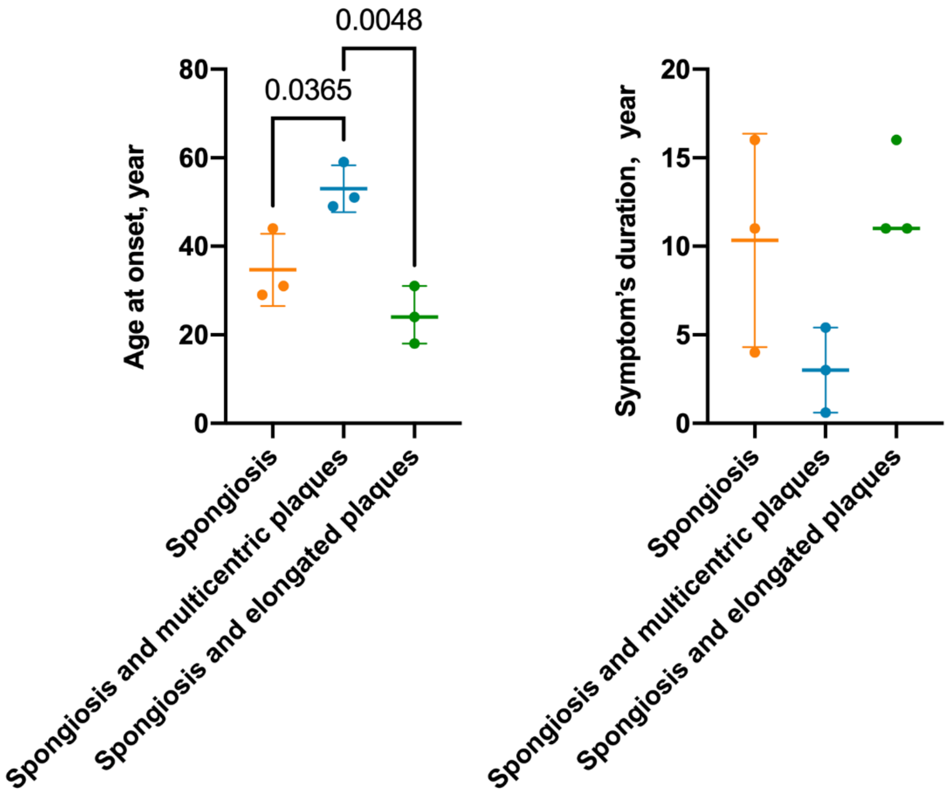

Figure S1. Comparison of age at onset and duration of symptoms in patient groups demarcated by pathological changes.

Table S1. Clinical and auxiliary characteristics of all known 17 patients carrying 7-OPRI.

Number	Year	Country	Gender	Family History	Age at Onset (Years)	Duration(Years)	First Symptom of Disease	Cognitive Dysfunction	Psychiatric Disturbances	Parkinsonism	Cerebellar Signs	Myoclonus	Pyramidal Signs	Speech Disorders	References
1	1991	USA	F	+	31	11	Psychiatric disturbances	+	+ (Mood change, indifference, confusion)	+	+		+	+	[8,10]
2			F	+	23	10	Psychiatric disturbances	+	+ (Abnormal behavior)	+	+	+	+	+	
3			F	+	28	13	Ataxia (abnormal gait)	+	+ (Euphoria)	+	+	+	+	+	
4	1992	Japan	NA	+	NA	7	Cognitive dysfunction	+		+					[9]
5	2000	Belgium	M	+	32	7	Cognitive dysfunction	+		+					[11]
6			M	+	24	11	Cognitive dysfunction	+	+ (Psychosis)	+	+	+			
7			F	+	31	11	Ataxia	+	+ (Depression/ mood change)		+		+		
8	2003	Australia	NA	+	29	16	Psychiatric disturbance and	+	+ (Abnormal behavior)	+					[12]

							cognitive dysfunction								
9	2007	Italy	M	-	18	16	Cognitive dysfunction and psychiatric disturbance	+	+ (Depression, psychosis, bipolar disorder)		+				[13,14,17]
10	2008	China	F	+	44	4.2	Cognitive dysfunction	+			+	+	+		[15,16]
11	2011	Netherlands	F	+	50	0.7	Cognitive dysfunction	+	+ (Emotional lability, anxiety attacks)				+	+	[7]
12			M	+	49	3	Cognitive dysfunction	+	+ (Emotional lability, apathetic)	+					
13			M	+	59	0.6	Ataxia	+	+ (Apathetic)	+	+	+			
14			M	+	51	5.4	Ataxia, cognitive dysfunction and psychiatric disturbance	+	+ (Depression, panic attacks)	+	+	+			
15	2022	China	NA	+	40s	>0.6	Cognitive dysfunction	+	+ (Mood change, abnormal behavior)	+	+	+	+		Our case

16		NA	+	60s	5.5	Cognitive dysfunction	+	+ (Mood change, abnormal behavior)	+	+		+	+	Our case
17		NA	+	50s	1.5	Cognitive dysfunction	+			+	+			Our case

Table S1. Continued.

Number	Visual Signs	Mutism	Seizure	PSWCs on EEG	CSF 14-3-3 Protein	CSF Tau Protein	CSF RT-QuIC	Hyperintensity on MRI	Condon 129	Neuropathology	Immunoblot	Mutation	Misdiagnose	References
1			+	- (Diffuse slowing)					Cis-M	Spongiosis, gliosis, neuronal loss		R2c–R3–R2–R3–R2–R3–R2–R3g	Schizophrenia	[8,10]
2		+		- (Diffuse slowing)					Cis-M	Mild gliosis and neuronal loss (no spongiosis)				
3		+		- (Paroxysmal episodes of slow wave spikes)					Cis-M					
4									NA	Kuru-like plaques in the cerebellum		R3–R2–R2–R2–R3g–R2–R2		[9]
5									Cis-M			NA	Multiple sclerosis	[11]
6										Spongiosis, gliosis, neuronal loss, elongated				

								cerebellar plaques cerebellar cortex				
7		+					Cis-M(MM)	Spongiosis, gliosis, neuronal loss, elongated cerebellar plaques cerebellar cortex				
8		+	- (Diffuse slowing)				Cis-M(MV)	Spongiosis, gliosis, neuronal loss, non-linear granular deposits cerebellar cortex	Type 1	R3–R2–R3–R2–R2–R2–R2	Huntington's disease	[12]
9	+		- (Diffuse slowing)	-	-	- (MRI revealed widespread cortical brain atrophy)	Cis-M(MM)	spongiform degeneration, cortical PrP plaques, and elongated PrP formations in the cerebellum.	type 1 in the cerebral cortex and mixed types 1 and 2 in the cerebellum	R3–R2–R2–R3g–R2–R2–R2	Psychosis and bipolar disorder	[13,14,17]
10	+		+			- (no remarkable changes)	Cis-M(MM)	Spongiosis, gliosis, neuronal loss	Type 1	R2–R2–R2–R3g–R2–R3g–R2a		[15,16]
11							NA			NA		[7]
12			- (Non-specific changes)				NA	Spongiosis +++, gliosis, neuronal loss, multicentric plaques	Type 1	NA		

								(cerebellum ++, cortex +)				
13		+				+ (mild symmetric hyperintense lesions in the basal ganglia)	Cis-V(MV)	Spongiosis +, gliosis, neuronal loss, multicentric plaques (cerebellum +++, cortex ++)	Type 1 and 8 kDa	R2–R2–R2–R2–R3g–R2–R2		
14		- (Slowing of background activity)				- (No hyperintense lesion)	Cis-V(VV)	Spongiosis+++, gliosis, neuronal loss, multicentric plaques (cerebellum++, cortex +)		R2–R2–R2–R2–R3g–R2–R2		
15	+	- (non-specific changes)	+	+	+	+ (Cortical ribbon sign)	Cis-M(MM)			R2-R2-R2-R3g-R2-R3g-PHGGGWEQ		Our case
16						- (Brain atrophy)	Cis-M(MM)			R2-R2-R2-R3g-R2-R3g-PHGGGWEQ	Alzheimer disease	Our case
17	+	- (Diffuse slowing)	+	+	+	+ (Cortical ribbon sign)	Cis-M(MM)			R2-R2-R2-R3g-R2-R3g-PHGGGWEQ		Our case

CSF: cerebrospinal fluid; EEG: electroencephalogram; MRI: magnetic resonance imaging; OPRIs: octapeptide repeat insertions; PSWCs: periodic sharp wave complexes; RT-QuIC: real-time quaking-induced conversion.
